# New Method of Treating Ulcers

**Published:** 1873-06

**Authors:** 


### ARTICLE XI.

### *New Method of Treating Ulcers.*

Dr. Philip Cowen, makes some interesting suggestions with reference to the treatment of ulcers. He regards an ulcer as a local asthenia of the skin and parts beneath, a local weakness and loss of plasticity, a brittleness where softness, elasticity and pliancy, yet strength, should exist, a local tendency to degeneration and death. Knowing that an ulcer has power of absorbing matters applied to its surface, he availed himself of this property by applying, locally, matters having nutritive powers, so that the skin might be nourished locally at the weakened and degenerate spots, by taking up such matters as would nourish its weakness, and convert its brittleness into a plastic and healing state. To apply this theory he made a paste of the following substances: Flour, four ounces; powder of acacia, one ounce; chalk, two drachms; cold water, one pint. These were boiled gently for a minute, and then allowed to cool. It then was thinned, by the addition of water, till it could be readily spread over the ulcer by means of a brush. The application was made three or four times daily, care being taken to keep the paste perfectly fresh and sweet.

Seventeen cases are reported, in which more or less rapid healing took place. These all occurred in a work house, under anything but favorable conditions. The Dr. observes:

1. That islands of skin arise in some cases in the center of the ulcer as well as from the circumference.

2. For some days after applying the paste there is a large increase in the ulcer secretion, but it soon becomes normal.

3. The remedy is painless.

4. The cicatrix is peculiarly healthy and strong in appearance.—*Lancet. Review of Med. & Pharmacy.*

